# The constrictive consequences of pericardial calcifications

**DOI:** 10.1002/ccr3.3116

**Published:** 2020-08-06

**Authors:** Anastasia D. Egorova, Martin J. Schalij, Philippine Kiès

**Affiliations:** ^1^ Department of Cardiology Heart Lung Center Leiden University Medical Center Leiden The Netherlands

**Keywords:** congenital heart disease, constrictive pericarditis, dip and plateau pattern, square root sign

## Abstract

A 53‐year‐old man presented with symptomatic severe pulmonary valve regurgitation. He underwent a diagnostic catheterization. A heavily calcified pericardium and the pressure tracings illustrate typical features of constrictive pericarditis physiology, including the "square root sign." This condition is important to recognize given the progressive nature and poor prognosis if untreated.

## Graphical abstract

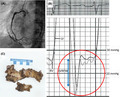

A 53‐year‐old male with a history of surgical pulmonary commissurotomy at the age of 12 presented with progressive shortness of breath, right‐sided pleural effusion, and peripheral edema. He was in atrial fibrillation with adequate rate control. Echocardiogram showed normal systolic biventricular function and severe pulmonary regurgitation (dilated right ventricle with signs of volume overload; wide origin color flow regurgitation jet, dense continuous wave signal, steep deceleration [pressure half time 80 ms]). Patient responded well to diuretics and underwent a successful cardioversion, after which rhythm control strategy was pursued. A diagnostic cardiac catheterization was performed in the workup for a pulmonary valve replacement. How should the findings shown in Figure 1 be interpreted?

**Figure 1:**
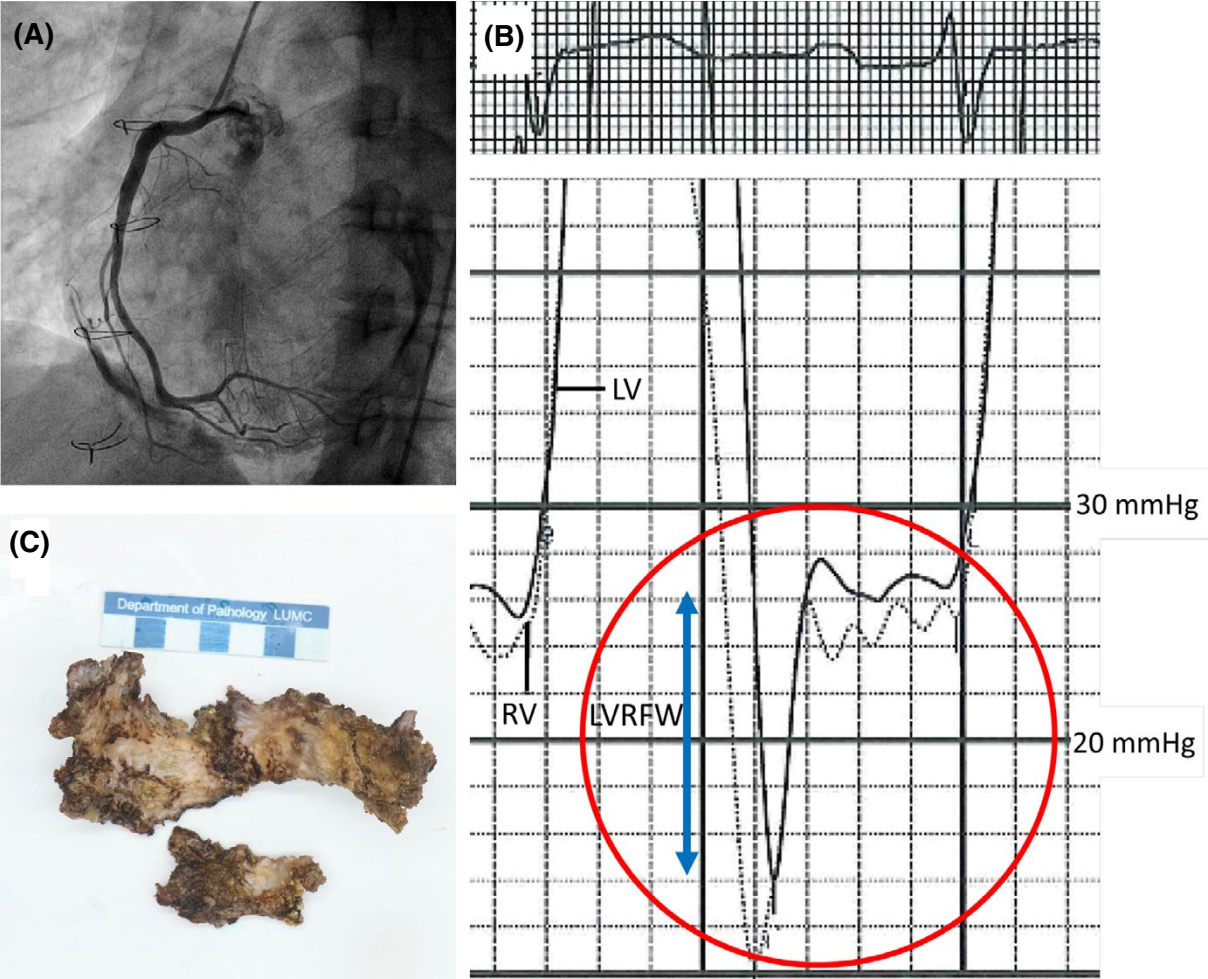
A, Selective coronary angiography of the right coronary artery showing no obstructive lesions, but revealing a remarkable radio‐opaque aspect of the calcified pericardium. B, Simultaneous right and left ventricular pressure tracings showing diastolic pressure equalization in both chambers and the "square root sign" (encircled in red). Left ventricular rapid‐filling wave (LVRFW) is accentuated (blue arrow). C, Pathological specimen of the surgically removed heavily calcified pericardium

The diagnostic catheterization revealed constrictive pericarditis physiology. Extensive pericardial calcifications were evident during X‐ray exposure. Mean right atrial pressure was significantly elevated at 12 mm Hg (SvO_2_ 69%). Simultaneous right (SvO_2_ 67%) and left (SaO_2_ 98%) ventricular pressure tracings revealed diastolic pressure equilibration (23‐26 mm Hg) and the pathognomonic "dip and plateau pattern," also known as the "square root sign".^1^ Left ventricular rapid‐filling (LVRFW) wave was accentuated and measured ±10mm Hg. LVRFW > 7mm Hg is representative of the increased early diastolic ventricular filling and is a sensitive marker for constrictive physiology.^2^ Mean pulmonary artery pressure was 25 mm Hg. Patient had no obstructive coronary artery disease. He underwent a resection of the heavily thickened and calcified pericardium and a pulmonary valve replacement with a homograft. The postoperative recovery period was uneventful, and the patient is currently symptom‐free 6 months after the operation.

## CONFLICT OF INTEREST

The authors declare that they have no competing interests.

## AUTHOR CONTRIBUTIONS

All authors were involved in analysis and interpretation of the diagnostic data described in the manuscript, were involved in writing and editing of the manuscript, and have approved the manuscript for submission in its current form.
